# Atypical Neural Responses During Face Processing in Female Adolescents With Conduct Disorder

**DOI:** 10.1016/j.jaac.2014.02.009

**Published:** 2014-06

**Authors:** Graeme Fairchild, Cindy C. Hagan, Luca Passamonti, Nicholas D. Walsh, Ian M. Goodyer, Andrew J. Calder

**Affiliations:** a University of Southampton, UK; b University of Cambridge, UK; c Consiglio Nazionale delle Ricerche, Istituto di Bioimmagini e Fisiologia Molecolare, Italy; d University of East Anglia, UK; e Medical Research Council Cognition and Brain Sciences Unit, Cambridge, UK

**Keywords:** CD, CU traits, females, face processing, fMRI

## Abstract

**Objective:**

Conduct disorder (CD) in females is associated with negative adult outcomes including mental health problems and personality disorders. Although recent neuroimaging studies have reported changes in neural activity during facial emotion processing in males with CD or callous-unemotional (CU) traits, there have been no neuroimaging studies specifically assessing females with CD. We addressed this gap by investigating whether female adolescents with CD show atypical neural activation when processing emotional or neutral faces.

**Method:**

We acquired functional magnetic resonance imaging (fMRI) data from 20 female adolescents with CD and 20 female control participants while they viewed angry, sad, and neutral faces.

**Results:**

An omnibus group (CD, control) by facial emotion (angry, sad, neutral) analysis of variance (ANOVA) revealed main effects of facial emotion in superior temporal cortex, fusiform gyrus, ventrolateral prefrontal cortex and insula, and main effects of group in medial orbitofrontal cortex (OFC) and right anterior insula. Female participants with CD showed reduced medial OFC and increased anterior insula responses relative to healthy controls. There were no significant group × facial emotion interactions. Lifetime CD symptoms were negatively correlated with amygdala, superior temporal cortex, fusiform gyrus, and dorsolateral prefrontal cortex activity for the contrast “all-faces versus fixation.” CU traits were negatively correlated with fusiform gyrus activity for the contrast sad versus neutral faces.

**Conclusion:**

Females with CD showed atypical neural activation during the processing of all facial expressions, irrespective of valence. Our results demonstrate that severity of CD symptoms and CU traits is important in explaining abnormal patterns of neural activity.

Conduct disorder (CD) is characterized by a pervasive pattern of antisocial and violent behavior in which the rights of others are violated.^1^ CD is one of the most common disorders in adolescent females^2^ and is associated with an increased risk of developing antisocial or borderline personality disorder, substance dependence, depression, and physical health problems in adulthood.^3^, ^4^, ^5^, ^6^ Despite this negative prognosis, we know relatively little about the neurobiological mechanisms underlying CD in females, as there have been few neuropsychological studies of this group, and as functional magnetic resonance imaging (fMRI) studies of CD have been largely restricted to males. Of the previous 26 fMRI studies of CD, 17 have included only males, and the remaining 9 studies recruited mixed samples containing only a small number of females, resulting in an underrepresentation of females with CD (442 males versus 41 females pooled across 26 studies; Table S1, available online, provides references). Critically, none of these studies investigated brain activity in females with CD specifically.

To address this gap in the literature, we investigated neural responses to emotional and neutral facial expressions in female adolescents with CD relative to healthy controls. An earlier behavioral study found impaired recognition of  facial expressions of anger and disgust in female adolescents with CD, and an additional impairment in sadness (but not fear) recognition in females with CD and psychopathic traits.^7^ Similar deficits in anger and disgust recognition were observed in males with CD, ^8^ indicating that CD in both sexes is associated with difficulties in processing these emotions. In contrast, psychopathic traits were associated with deficits in both sadness and fear recognition in males with CD.^8^ To follow up these behavioral findings and characterize the underlying neural processes, we previously conducted an fMRI study and observed reduced amygdala, anterior insula, orbitofrontal cortex (OFC), and anterior superior temporal cortex responses to emotional versus neutral faces in male adolescents with CD.^9^

On the basis of these earlier results, we predicted that female adolescents with CD would show atypical neural responses when processing angry or sad relative to neutral facial expressions. Specifically, we predicted that females with CD would show reduced activity in regions involved in social cognition and emotion processing, such as the amygdala, anterior insula, OFC and superior temporal cortex, during the processing of negative facial expressions.^10^, ^11^ This would be demonstrated by significant interactions between group and facial emotion in these regions, in which healthy controls would show greater neural responses to angry and sad faces than neutral faces. Meanwhile, participants with CD would show weaker differentiation between these facial expressions. However, it was also possible that females with CD would show increased neural responses to neutral faces, as we previously found that male adolescents with CD showed increased amygdala and insula responses to neutral faces.^9^

Our second aim was to examine relationships between brain activity and severity of CD, as quantified by number of CD symptoms. We predicted that CD symptoms would be negatively correlated with amygdala, anterior insula, OFC and superior temporal cortex activity, given previous research in males showing negative relationships between activity in these regions and CD symptoms^9^ or aggressive behavior.^12^

The presence of CU traits (such as emotional detachment) is thought to delineate a particularly severe and persistent form of CD.^13^ Given the importance of callous-unemotional (CU) traits for understanding heterogeneity within antisocial behavior,^14^ our final aim was to investigate whether CU traits would modulate neural activity during facial emotion processing. The Integrated Emotion Systems (IES) model^15^ proposes that distress cues, such as sad or fearful facial expressions, play a critical role in the socialization process. According to this model, typically developing children find distress cues aversive, so they learn to stop engaging in aggressive behaviors that elicit such cues. Individuals with CU traits are proposed to be insensitive to distress cues, which disrupts their socialization, rendering them at increased risk for instrumental aggression. The IES model therefore predicts that CU traits would be associated with impaired recognition of sad and fearful expressions, along with reduced neural responses to these facial expressions. Previous research has provided evidence for selective or disproportionate impairments in sadness and fear recognition in individuals with CU traits^8^, ^16^, ^17^ (although see Dawel *et al*.^18^ for a meta-analysis showing pervasive emotion recognition deficits in psychopathy). However, with the exception of 1 study showing impaired sadness recognition in female adolescents with CD and CU traits^7^ and a study reporting deficits in sadness recognition in female psychopaths,^19^ most previous studies of CU traits or psychopathy have either focused on males alone or have included small numbers of females.^16^, ^17^, ^18^ fMRI studies have shown reduced amygdala responses to fearful facial expressions in male children with conduct problems and CU traits^20^ and in a group of adolescents with CU traits and disruptive behavior disorder diagnoses who were predominantly male.^21^ Studies in adults have shown reduced amygdala or fusiform gyrus responses to fearful faces in males with psychopathy.^22^, ^23^ However, no comparable data exist on the effects of CU traits on neural activation in females. To further investigate the IES model, we assessed whether CU traits were associated with reduced brain activation during the processing of sad facial expressions. Sadness, rather than fear, was selected, given previous behavioral results showing that CU or psychopathic traits were associated with impaired recognition of sadness but not fear in females,^7^, ^19^ and on the basis of a meta-analysis showing that CU traits are most strongly linked with deficits in sadness recognition.^18^ We predicted that CU traits would be negatively correlated with amygdala, anterior insula, OFC and fusiform gyrus responses to sad versus neutral expressions.

## Method

### Participants

Twenty-two female adolescents with CD were recruited from schools, pupil referral units, and the Cambridge Youth Offending Service. All participants gave written informed consent to participate in the study, which was approved by the local National Health Service research ethics committee. Exclusion criteria for the CD group included the following: IQ <80, as estimated using the Wechsler Abbreviated Scale of Intelligence,^24^ or presence of a pervasive developmental disorder (e.g., autism). A healthy control group (HC; no history of CD/oppositional defiant disorder [ODD] or current psychiatric illness) of 22 female adolescents, matched in age, handedness, ethnicity, and performance IQ, was recruited from schools and colleges. This sample overlaps substantially (95%) with the female sample included in an earlier structural MRI study.^25^

All participants were assessed for CD, ODD, attention-deficit/hyperactivity disorder (ADHD), major depressive disorder (MDD), generalized anxiety disorder (GAD), obsessive-compulsive disorder (OCD), posttraumatic stress disorder (PTSD), and substance dependence, using the Schedule for Affective Disorders and Schizophrenia for School-Age Children (K-SADS).^26^ Diagnostic interviews were carried out separately with participants and caregivers. The majority (n = 17) of the females with CD had the adolescence-onset form of CD (i.e., onset of CD symptoms only after age 10 years^1^).

Both CU and psychopathic traits were assessed using the Callous-Unemotional dimension subscale and the total score of the self-report Youth Psychopathic traits Inventory (YPI),^27^ respectively. CU traits data were also obtained from parents using the Inventory of Callous-Unemotional traits (ICU).^28^ The Adolescent Alcohol and Drug Involvement Scale (AADIS) measured alcohol and substance use.^29^ Handedness was assessed using the Edinburgh Handedness Inventory.^30^ Finally, socioeconomic status (SES) was quantified using the A Classification Of Residential Neighbourhoods (ACORN) geodemographic tool (http://www.caci.co.uk/acorn-classification.aspx).

To control for menstrual cycle phase effects on brain activity,^31^ all participants with CD and the majority of the healthy control participants were scanned in the mid-follicular phase of the cycle (i.e., within 5–10 days of menstruation onset), as determined by self-report.

### fMRI Task

**Figure S1:**
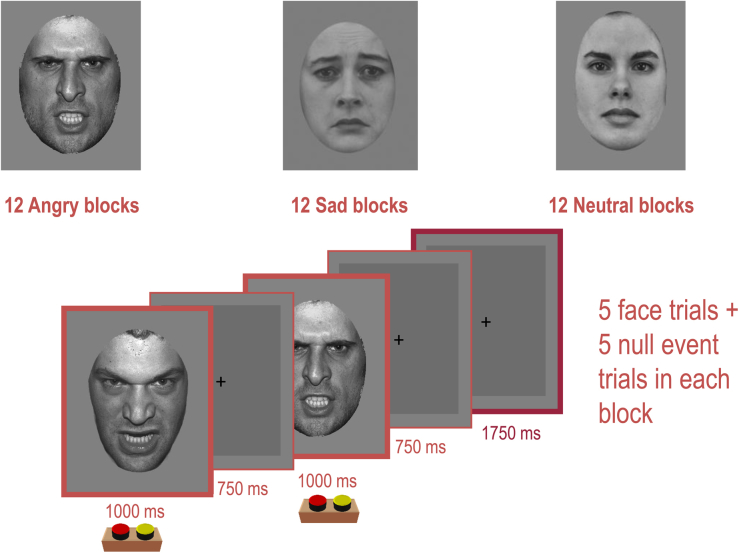
Schematic representation of the functional magnetic resonance imaging (fMRI) task and examples of the facial stimuli used in the experiment. Note: All participants were shown gray-scale photographs of angry, sad, or neutral facial expressions (12 epochs of each) intermixed with null events (fixation cross) within 17.5-second epochs. In each face trial, participants had to indicate whether the face was male or female by pressing either the left or right key on a button box. Each trial lasted 1,750 milliseconds, and total scanning time was 10 minutes 30 seconds.

Participants categorized the gender of gray-scale photographs of angry, sad, and neutral faces (half female) posed by 30 different identities, by pressing either the left button on a button box to indicate that the face was male or the right button to indicate the face was female (Figure S1, available online). The faces were selected from 2 stimulus sets^32^, ^33^ on the basis of emotional ratings from an independent sample.^34^ In a mixed design, stimuli were presented in 17.5-second epochs containing 5 faces from the same category (angry, sad, or neutral) interspersed with 5 null events (fixation cross). Each face trial comprised a 1,000-millisecond presentation of a face followed by a fixation cross (750 milliseconds). Null events involved a 1,750-millisecond presentation of the fixation cross. The stimuli presented during each epoch were pseudo-randomized with respect to trial type (faces or null events) and facial gender and identity; no more than 3 consecutive trials were of the same trial type. This pseudo-randomization enhanced design efficiency while ensuring that the stimulus onsets and valences were unpredictable for naive observers.^9^, ^35^ Twelve epochs of each expression were presented (60 angry, 60 sad, and 60 neutral faces). Reaction times (RTs) and accuracy were recorded throughout the experiment, which lasted 10 minutes 30 seconds. Subjective ratings of the emotional intensity of the stimuli were also obtained after scanning (participants rated each of the facial expressions used in terms of anger and sadness intensity, using a scale from 1 = not at all to 9 = very [angry or sad]).

### Image Acquisition and Preprocessing

MRI scanning was performed on a 3-Tesla Siemens Tim Trio with a head coil gradient set at the Medical Research Council (MRC) Cognition and Brain Sciences Unit at University of Cambridge. Whole-brain data were acquired with echo-planar T2*-weighted imaging (EPI), sensitive to blood-oxygenation-level–dependent (BOLD) signal contrast (32 axial slices, 3-mm thickness; repetition time = 2,000 milliseconds; echo time = 30 milliseconds; voxel size = 3 × 3 × 3 mm). Data were analyzed using SPM5 (www.fil.ion.ucl.ac.uk/spm/). EPIs were sinc-interpolated in time to correct for slice time differences and were realigned to the first scan by rigid body transformations to correct for head movements. The mean EPI was computed for each participant and inspected to ensure that no participants showed excessive signal dropout in medial temporal and OFC regions. EPIs were co-registered and normalized to the T1 standard template in Montreal Neurological Institute (MNI) space using linear and nonlinear transformations, and smoothed with an 8-mm Gaussian kernel of full width at half maximum.

### fMRI Analyses

For each participant, a general linear model (GLM) assessed regionally specific effects of task parameters on BOLD activation.^36^ The model included experimental factors (angry, sad, neutral face trials, and fixation trials) and 6 realignment parameters as effects of no interest, to account for residual motion-related variance. Low-frequency signal drift was removed using a high-pass filter (cut-off at 128 seconds) and an autoregressive modelling [AR(1)] of temporal autocorrelations was applied.

We ran a group (CD, control) × facial emotion (angry, sad, neutral) analysis of variance (ANOVA) to investigate for main effects of group or emotion and interactions between these factors. To follow up the main effects of group, we generated contrast images for “all faces versus fixation” and included these in regression analyses to assess whether individual differences in lifetime CD symptoms were correlated with neural activity. Given our a priori hypothesis that CU traits would be associated with reduced neural responses to sad facial expressions, we assessed whether CU traits were correlated with neural activity for the contrast sad versus neutral faces by using regression analysis. We also repeated the group-based analyses in SPM5, including lifetime or current ADHD symptoms or lifetime MDD diagnoses as covariates to examine the influence of these variables on the main findings.

Two approaches for thresholding second level maps were applied. First, to conduct whole-brain analyses, we applied a threshold of *p* < .05, false-discovery rate whole-brain correction.^37^ Second, in our a priori regions of interest (ROIs), the threshold used was *p* < .05, family-wise error correction for multiple comparisons in small volumes (i.e., small volume correction [SVC]).^38^, ^39^ The amygdala, ventromedial prefrontal cortex, anterior insula, OFC, fusiform gyrus, and superior temporal gyrus were defined as our ROIs, given that several previous fMRI studies of CD have shown group differences in these regions.^9^, ^12^, ^20^, ^21^, ^40^, ^41^, ^42^ All ROIs were anatomical regions defined using the “aal.02” atlas for automated anatomical labelling.^43^ For completeness and to aid future meta-analyses, we also report all brain regions that were significant at *p* < .001, uncorrected, ≥10 contiguous voxels, in Table 1, Table 2, Table 3.

**Table 1:** Demographic and Clinical Characteristics of the Female Participants

Characteristics	Group		Group Comparisons (*p* values)
	HC (n = 20)	CD (n = 20)	
Age (y)	17.62 ± 0.64	16.97 ± 1.52	.09
Full-scale IQ	104.60 ± 8.72	99.65 ± 8.06	.07
Performance IQ	105.00 ± 10.85	101.50 ± 9.99	.30
Handedness (R/L)	20/0	19/1	.50
No. of current *DSM-IV* diagnoses			
ADHD	0	3	.23
Substance abuse	0	2	.49
Panic disorder	0	1	.99
Number of past *DSM-IV* diagnoses			
ADHD	0	4	.11
MDD	3	9	.08
Substance abuse	0	4	.11
PTSD	0	1	.99
No. of symptoms			
Current CD	0.13 ± 0.34	2.70 ± 2.58	<.001
Lifetime CD	0.38 ± 0.62	7.70 ± 2.30	<.001
Aggressive CD	0.06 ± 0.25	2.70 ± 1.08	<.001
Current ADHD	1.55 ± 1.88	6.15 ± 3.47	<.001
Lifetime ADHD	1.90 ± 2.20	8.30 ± 3.85	<.001
Total psychopathic traits (total YPI)	1.65 ± 0.35	2.03 ± 0.41	.006
YPI CU traits subscale	0.53 ± 0.11	0.60 ± 0.13	.069
Inventory of CU traits	17.81 ± 8.04	28.78 ± 14.11	.010
SES (ACORN)			
Wealthy achievers	9	4	
Urban prosperity	0	4	
Comfortably off	6	3	.067
Moderate means	1	1	
Hard-pressed	4	8	
Ethnicity			
White	20	20	1.00
Nonwhite	0	0	
fMRI task performance			
Accuracy, %			
Angry	91 ± 7	89 ± 6	
Sad	96 ± 3	94 ± 5	.25
Neutral	93 ± 5	92 ± 5	
RTs, ms			
Angry	641 ± 68	635 ± 43	
Sad	635 ± 70	633 ± 45	.98
Neutral	635 ± 69	644 ± 45	

Note: Data are presented as mean ± SD or number in each group. ACORN = A Classification Of Residential Neighbourhoods; ADHD = attention-deficit/hyperactivity disorder; CD = conduct disorder; CU = callous-unemotional; fMRI = functional magnetic resonance imaging; HC = healthy control; L = left; MDD = major depressive disorder; PTSD = posttraumatic stress disorder; R = right; RT = reaction time; SES = socioeconomic status; YPI = Youth Psychopathic Traits Inventory.

**Table 2:** Coordinates and Cluster Sizes for the Main Effects of Emotion, Group, and Group × Emotion Interactions

Cerebral Region	Hemisphere	Local Maxima, F	No. of Significant Voxels in Cluster	MNI Coordinates		
				X	Y	Z
Main Effects of Facial Emotion						
Superior temporal gyrus/fusiform gyrus	R	23.58^a^	979	52	−38	6
Fusiform gyrus	L	17.87^a^	127	−42	−40	−16
	R	10.84^a^	73	44	−42	−16
Ventrolateral prefrontal cortex	R	13.56^a^	131	52	34	0
Middle temporal gyrus	L	12.87^a^	373	−52	−54	4
Precentral gyrus	R	11.15^a^	138	30	−26	64
Posterior insula	R	10.34^a^	73	40	−12	20
Cuneus	L	9.04^a^	42	−16	−78	4
Anterior insula	R	8.57^a^	16	36	26	6
Main Effects of Group						
Medial orbitofrontal cortex	R	11.46^b^	11	12	58	−2
Anterior insula	R	12.08^b^	52	42	16	8
Precentral gyrus	L	17.99	163	−28	−12	58
Precentral gyrus	R	18.12	201	28	−12	52
Cuneus	L	18.12	128	−10	−102	2
	R	11.95	27	10	−76	16
Medial frontal gyrus	R	15.33	87	10	12	52
Middle temporal gyrus	R	13.89	61	46	−58	−2
Cerebellum/fusiform gyrus	R	12.18	39	24	−82	−20
Lingual gyrus	R	11.86	32	28	−70	−2
Postcentral gyrus	R	11.51	16	46	−26	42
Group × facial emotion interactions						
No significant activations						

Note: Unless otherwise indicated, all regions were significant at *p* < .001, uncorrected, ≥10 contiguous voxels. L = left; MNI = Montreal Neurological Institute; R = right.

a *p* < .05, false-discovery rate whole-brain correction.

b *p* < .05, family-wise error small-volume correction.

**Table 3:** Coordinates and Cluster Sizes for Negative Correlations Between Lifetime Conduct Disorder Symptoms and Neural Activation for the Contrast “All Faces Versus Fixation”

Cerebral Regions	Hemisphere	Local Maxima, Z	No. of Significant Voxels in Cluster	MNI Coordinates		
				x	y	z
Amygdala	L	3.13^b^	14	−20	0	−24
Superior temporal gyrus	L	4.73^a^^,^^b^	513	−52	−4	−8
Superior temporal gyrus	R	3.98^a^^,^^b^	321	42	−6	−14
Dorsolateral PFC	L	4.59^a^	259	−32	36	42
Fusiform gyrus	L	3.69^a^^,^^b^	167	−28	−54	−12
Fusiform/hippocampus	R	4.27^a^^,^^b^	108	40	−14	−26
Fusiform gyrus	R	3.68^a^^,^^b^	58	24	−48	−14
Superior occipital cortex	L	4.10^a^	73	−22	−76	40
Middle occipital cortex	L	3.71^a^	53	−40	−78	6
Supplementary motor area	L	3.57	29	−10	−4	56
Parahippocampal gyrus	R	3.33	10	14	−12	−22

Note: Unless otherwise indicated, correlations met the criteria of *p* < .001, uncorrected, for ≥10 contiguous voxels. L = left; MNI = Montreal Neurological Institute; PFC = prefrontal cortex; R = right.

a *p* < .05, false-discovery rate whole-brain correction.

b *p* < .05, family-wise error small-volume correction.

## Results

Three participants (2 with CD and 1 control) were excluded because of excessive head movements (>2 mm) during scanning, and a further control participant was excluded because of the presence of neurological abnormalities as indicated by an accompanying structural scan.

### Demographic and Clinical Variables

There were no significant group differences in full-scale IQ, age, or SES (Table 1). The groups were matched in ethnicity, handedness, and performance IQ. As in our previous research, the female CD participants had higher levels of psychopathic and CU traits relative to controls. The participants with CD also reported more CD and ADHD symptoms and lifetime MDD diagnoses than did the healthy control participants.

### Behavioral Results

Accuracy and correct RTs during the gender discrimination task were entered into a 2 × 3 ANOVA assessing for effects of group and emotion. Neither measure showed a group effect (accuracy, F_1,38_ = 1.35, *p* = .25; RT, F_1,38_ = 0.001, *p* = .98) or a group × emotion interaction (accuracy, F_2,76_ = 0.11, *p* = .89; RT, F_2,76_ = 1.87, *p* = .16) (Table 1).

**Figure S2:**
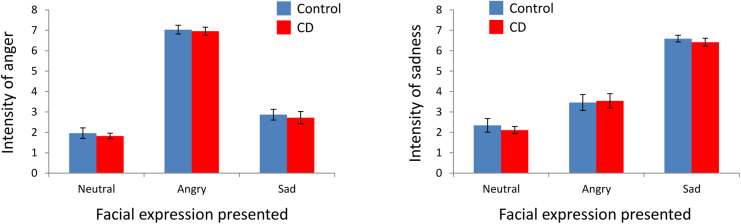
Ratings for anger and sadness intensity for the 3 classes of facial expressions used in the functional magnetic resonance imaging (fMRI) experiment, by group, as obtained after the fMRI scanning session. Note: Participants were asked to rate each facial expression in terms of anger intensity, and then rate each expression again in terms of sadness intensity. There were no group differences in ratings of emotion intensity or group by expression interactions in either the anger or the sadness rating conditions. CD = conduct disorder.

The rating data collected after scanning were entered into separate 2 × 3 ANOVAs assessing for effects of group and emotion on emotional ratings of sadness or anger intensity. There were no main effects of group (anger, F_1,38_ = 0.23, *p* = .63; sadness, F_1,38_ = 0.14, *p* = .71) or group × emotion interactions (anger, F_2,76_ = 0.02, *p* = .96; sadness, F_2,76_ = 0.25, *p* = .74; Figure S2, available online) on ratings of anger or sadness.

### fMRI Results

#### Main Effects of Emotion, Group, and Group × Emotion Interactions

**Figure S3:**
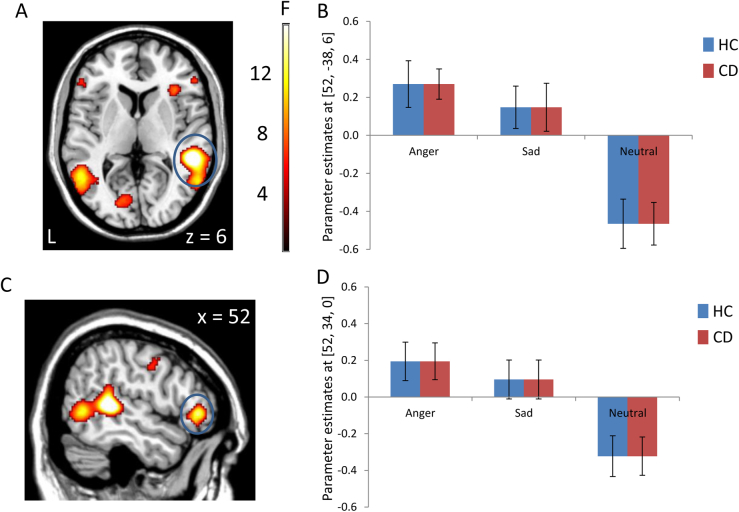
Main effects of facial emotion in the group × facial emotion analysis of variance. Note: Panel A shows the significant main effect of emotion in right posterior superior temporal gyrus/sulcus extending into fusiform gyrus (circled in blue), whereas Panel C depicts the main effect of emotion in right ventrolateral prefrontal cortex/inferior frontal gyrus (circled in blue). The color bar, which ranges from red to white, represents F statistics. Images are thresholded at *p* < .005, uncorrected, for display purposes only. Panels B and D display the plots of the data extracted from the peak voxels in superior temporal gyrus and ventrolateral prefrontal cortex, respectively. These plots show that both regions were most strongly activated by angry faces and least activated by neutral faces, with sad faces also tending to activate these regions to a greater degree than neutral faces. CD = conduct disorder; HC = healthy control.

We observed main effects of emotion in several regions involved in face processing and social cognition, such as superior temporal cortex, fusiform gyrus, ventrolateral prefrontal cortex, and anterior insula (all significant at *p* < .05, false discovery rate [FDR] whole-brain correction) (Table 2). These regions were more strongly activated by angry than neutral faces, with responses to sad faces falling at an intermediate level between angry and neutral (Figure S3, available online).

**Figure 1:**
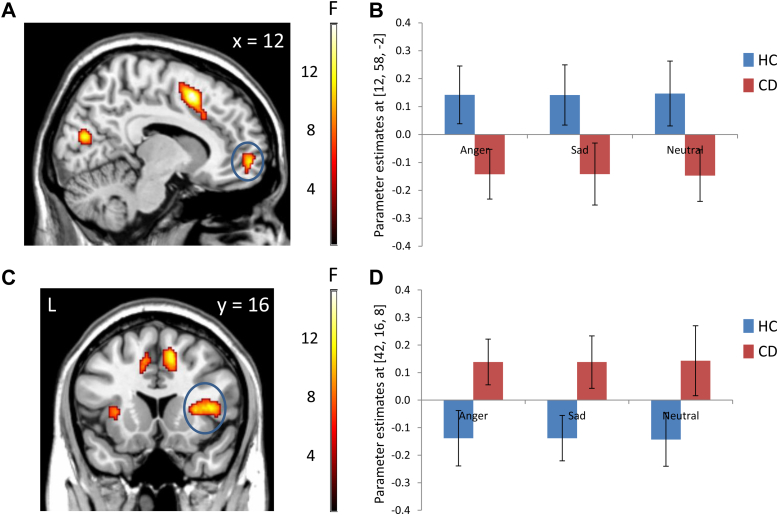
Main effects of group in the group × facial emotion analysis of variance. Note: The conduct disorder (CD) group showed significantly lower activation than the healthy control (HC) group in medial orbitofrontal cortex (panel A; circled in blue), whereas the CD group showed increased right anterior insula activity relative to the HC group (panel C; circled in blue). Color bars represent F statistics. The images are thresholded at *p*<0.005, uncorrected, for display purposes. Plots of the data extracted from the medial orbitofrontal cortex and right anterior insula are displayed in panels B and D, respectively. These plots indicate that the main effects of group were independent of facial expression valence. Coordinates and statistics for the group effects are provided in Table 2.

Main effects of group were observed in medial OFC and right anterior insula (both *p* < .05, SVC), together with several regions outside our ROIs (Table 2). Underlying these group effects, subsequent 2-sample t tests showed that the CD group displayed reduced medial OFC (*p* = .02, SVC) and increased anterior insula (*p* = .02, SVC) responses compared with healthy controls (Figure 1). There were, however, no significant group × emotion interactions. This suggests that the main effects of group in OFC and anterior insula were present across all facial expressions (including neutral), irrespective of valence.

#### Correlations Between Neural Activity and CD Symptoms Within the CD Group Only

**Figure 2:**
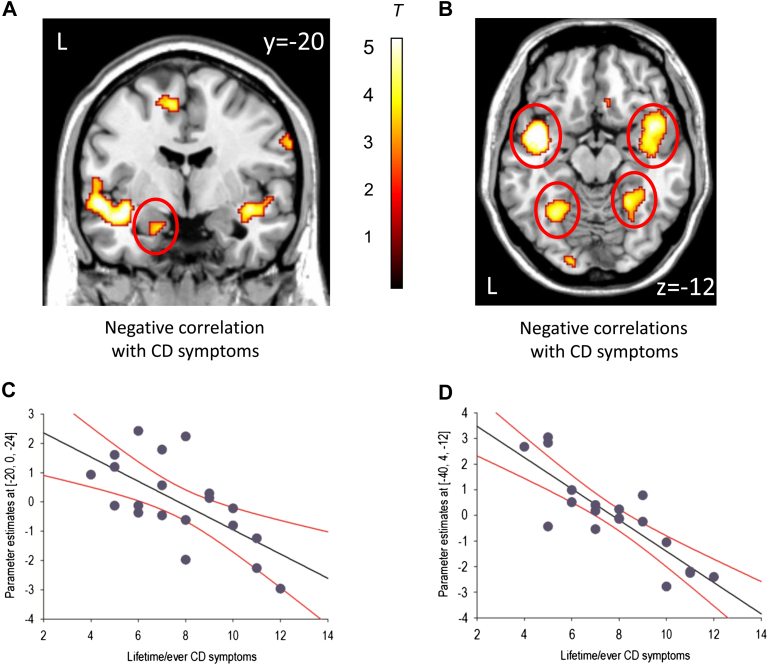
Negative correlations between lifetime conduct disorder (CD) symptoms and neural activity for the contrast “all faces versus fixation” within the CD group alone. Note: Panel A shows the negative correlation between lifetime CD symptoms and left amygdala activity (area circled in red), whereas Panel B shows the negative correlations between lifetime CD symptoms and bilateral anterior superior temporal gyrus and bilateral fusiform gyrus activity (circled in red). Coordinates and statistics for the correlations are provided in Table 3. The color bar represents T statistics. The images in panels A and B are thresholded at *p*<0.005, uncorrected, for display purposes. Panels C and D show scatter plots of the negative correlations between lifetime CD symptoms and left amygdala and left superior temporal gyrus activity, respectively. The regression lines are shown in black, whereas 95% confidence intervals are shown in red.

As the main effects of group were not modulated by facial emotion, to examine the effect of CD symptom severity, we generated contrast images for the contrast of “all faces versus fixation” and included these in our regression analyses. This was also the most balanced contrast, as there were an equal number of trials in each condition. We observed negative correlations between lifetime CD symptoms and activation in the left amygdala (*p* = .02, SVC), bilateral anterior superior temporal gyrus, bilateral fusiform gyrus, and left dorsolateral prefrontal cortex (all *p* < .05, FDR whole-brain correction; Figure 2 and Table 3 for coordinates). There were no significant positive correlations between lifetime CD symptoms and brain activity.

#### Correlations Between CU or Psychopathic Traits and Neural Responses to Sad Faces

**Figure 3:**
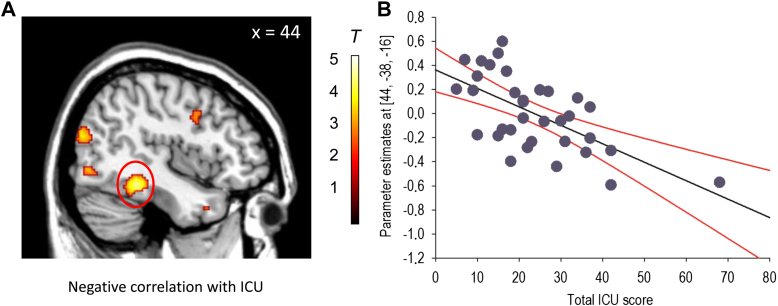
Negative correlation between parent-reported callous-unemotional (CU) traits and right fusiform gyrus activity for the contrast ‘sad versus neutral’ faces. Note: Panel A shows the negative correlation in sagittal format, whereas panel B shows a scatter plot of the negative correlation at the peak voxel in right fusiform gyrus (the regression line is shown in black, whereas 95% CIs are shown in red). The color bar represents T statistics. The image is thresholded at *p*<0.005, uncorrected, for display purposes. ICU = Inventory of Callous-Unemotional traits (parent-report).

As 1 of our hypotheses concerned the relationship between CU traits and sad faces, we performed additional regression analyses using contrast images generated for the comparison of sad versus neutral faces. Parent-reported CU traits were negatively correlated with right fusiform gyrus activity (*p* = 0.01, SVC) when considering the total female sample (i.e., healthy controls and participants with CD combined [N = 40]) (Figure 3). However, none of the other ROIs were correlated with parent-reported CU traits. There were also no significant correlations with self-reported CU or total psychopathic traits, and none of the ROIs were significantly correlated with parent-reported or self-reported CU or total psychopathic traits when considering the CD group alone.

#### Potential Confounds

The medial OFC and anterior insula group effects were still present at a trend level when controlling for lifetime diagnoses of major depressive disorder (medial OFC: *p* = .07, SVC; anterior insula: *p* = .10, SVC, respectively). The group effect in medial OFC was also present at a trend level when controlling for lifetime ADHD symptoms (*p* = .08, SVC) or current ADHD symptoms (*p* = .08, SVC), but the anterior insula finding was no longer present even as a trend in either case. However, when controlling for lifetime ADHD symptoms, an additional main effect of group emerged in right fusiform gyrus (*p* < .01, SVC). This effect was driven by participants with CD showing increased right fusiform gyrus activity relative to healthy controls.

## Discussion

The present study is, to our knowledge, the first fMRI study to investigate brain activity specifically in females with CD. Contrary to our hypothesis, we did not observe significant interactions between group and facial emotion such that female adolescents with CD showed weaker differentiation between negative and neutral facial expressions compared with healthy controls. Instead, we observed main effects of group in the medial OFC and anterior insula, which were present irrespective of valence. These findings were driven by females with CD showing reduced medial OFC and increased anterior insula activity relative to healthy controls. This pattern of results appears to differ from that observed in males with CD, who showed reduced neural responses to angry and increased responses to neutral facial expressions relative to healthy controls.^9^ Critically, behavioral data collected during scanning (i.e., reaction times and accuracy of gender discrimination) ensured that group differences in neural activation were not due to a failure to attend to the facial stimuli. In addition, the 2 groups gave similar affective ratings of the angry and sad facial stimuli when asked to rate them after scanning.

Rather than demonstrating an interaction between group and facial emotion that would indicate altered neural activity during the processing of negatively valenced facial expressions in CD, the present study found that females with CD showed reduced medial OFC responses to all classes of facial stimuli relative to controls. The medial OFC plays an important role in social cognitive processes, including facial expression decoding and mentalizing.^44^, ^45^, ^46^ The observation of increased right anterior insula responses to all facial expressions in females with CD is also interesting, given previous work reporting reduced anterior insula gray matter volume in females with CD.^25^ Although this structure–function relationship may appear to be contradictory, previous studies have found that reduced volume may lead to increased activity in the same region, or have shown no relationship between volume and functional activity.^47^ We also note that the group effect in the anterior insula was rendered nonsignificant when controlling for ADHD symptoms, suggesting that ADHD comorbidity may have contributed to this finding.

Consistent with our previous neuroimaging studies of males with CD^9^, ^48^ and studies showing dimensional relationships between aggression and brain structure in community samples of children,^49^ the present study demonstrates that severity of CD is an important dimension in explaining atypical patterns of neural activation. Lifetime CD symptoms were negatively correlated with activity in a network of brain regions involved in face processing and social cognition, including the amygdala, superior temporal cortex, dorsolateral prefrontal cortex, and fusiform gyrus.^10^, ^50^ These results provide further support for dimensional approaches to understanding externalizing disorders^51^ and suggest that neural abnormalities are most pronounced in females with severe forms of CD.

The third aim of the study was to investigate whether variation in CU traits was related to altered brain activation during face processing. We observed a negative relationship between CU traits and right fusiform gyrus activity for the contrast of sad versus neutral faces, consistent with previous work reporting reduced fusiform gyrus responses to distress cues in adult male psychopaths.^23^ However, consistent with our previous fMRI study of males with CD,^9^ there were no correlations between parent- or self-reported CU traits or total psychopathic traits and amygdala, insula, or OFC activation. Consequently, our findings differ from previous results showing reduced amygdala responses to distress cues in children with conduct problems and CU traits.^20^, ^21^ This disparity may be accounted for by the use of sad rather than fearful expressions, gender differences in the relationship between CU traits and reactivity to distress cues, or insufficient power to detect effects of CU traits.

The present study had several strengths. We controlled for menstrual cycle phase; the groups were well characterized in terms of psychiatric disorders and key demographic variables; data were collected from multiple informants on CU and psychopathic traits; and the fMRI task permitted the disaggregation of neural activity during emotion processing from face processing in general. We also collected data on gender discrimination performance while participants were in the scanner, thereby ensuring that all participants attended to the facial stimuli and engaged with the task. In terms of limitations, although high levels of psychiatric comorbidity in the CD group were to be expected given previous epidemiological work,^52^, ^53^ and although we controlled for comorbidity in our analyses, the fact that almost half of the female CD group had a current or past internalizing disorder (e.g., depression or anxiety) may have influenced our findings. It will therefore be important to recruit larger, noncomorbid CD samples in future research, although we note that there is a risk that such samples would be unrepresentative, as levels of psychiatric comorbidity are extremely high in females with CD. The cross-sectional nature of the study means that we cannot infer that the atypical neural activation observed plays a causal role in the etiology of CD. Future studies should adopt longitudinal designs to investigate whether atypical neural activation predicts the development of CD or resolves after successful treatment. We also note the possibility that females with CD may have shown atypical neural responses to any complex visual stimulus. Consequently, future studies should control for stimulus complexity by including complex but nonfacial stimuli or scrambled faces. Finally, although the gender discrimination data indicated that the participants with CD attended to the facial stimuli, their eye-scanning patterns may have differed from those of healthy controls.^54^ This may contribute to group differences in brain activity as has been found in studies of autism spectrum disorders.^55^ In future studies, eye-tracking data could be collected during fMRI data acquisition to address this issue.

Our observation of atypical medial OFC and anterior insula responses during face processing in females with CD provides further evidence that neurobiological factors may be involved in the etiology of severe antisocial behavior. These brain regions are involved in social cognitive processes, including the decoding of facial expressions and empathy, so our findings may help to explain previous results showing impaired facial emotion recognition in females with CD. These group differences in neural activity were observed across all facial expressions (including neutral), and so appear to be related to face processing in general rather than to emotion processing specifically. We also observed negative correlations between CD symptoms and neural activity in a network of regions involved in social cognition and also between CU traits and fusiform gyrus responses to sad facial expressions. These results support dimensional approaches to understanding externalizing disorders, as they suggest that atypical brain activity is most evident in those individuals with severe forms of CD or CU traits.

## Figures and Tables

**Table S1:** Sample Characteristics of Previous Functional Magnetic Resonance Imaging (fMRI) Studies of Conduct Disorder (CD), Conduct Problems, or Callous-Unemotional (CU) Traits

Authors, Year, Reference	Sample Description	No. of Male and Female CD Participants (Male:Female)	Task Used	Psychiatric Comorbidity
Sterzer *et al*. (2005)^1^	Childhood-onset CD	13:0	Passive viewing of IAPS pictures	8/13 Had comorbid ADHD; elevated anxiety and depression scores
Stadler *et al*. (2007)^2^	Childhood-onset CD	13:0	Passive viewing of IAPS pictures	8/13 Had comorbid ADHD; elevated anxiety and depression scores
Banich *et al*. (2007)^3^	Conduct problems and substance use	12:0	Stroop task	2/12 Had comorbid ADHD
Marsh *et al*. (2008)^4^	Disruptive behavior disorders + CU	7:5	Fear, anger, and neutral face processing	7/12 Had comorbid ADHD
Herpertz *et al*. (2008)^5^	Childhood-onset CD	22:0	Passive viewing of IAPS pictures	16/22 Had comorbid ADHD; elevated anxiety and depression scores
Finger *et al*. (2008)^6^	Disruptive behavior disorders + CU	9:5	Probabilistic reversal learning	10/14 Had comorbid ADHD
Jones *et al*. (2009)^7^	Conduct problems + CU	17:0	Fear and neutral face processing	Not assessed
Decety *et al*. (2009)^8^	Childhood-onset CD	8:0	Empathy for pain task	7/8 Had comorbid ADHD
Rubia *et al*. (2008)^9^	Childhood-onset CD	13:0	Stop task	None
Rubia *et al*. (2009a)^10^	Childhood-onset CD	14:0	Rewarded continuous performance task	None
Rubia *et al*. (2009b)^11^	Childhood-onset CD	13:0	Simon interference task	None
Gatzke-Kopp *et al*. (2009)^12^	Mixed externalizing disorders	19:0	Monetary incentive delay	16/19 Had ADHD diagnoses; elevated depression scores
Bjork *et al*. (2010)^13^	Mixed externalizing disorders	9:3	Monetary incentive delay	3/12 Had comorbid ADHD; elevated internalizing scores
Passamonti *et al*. (2010)^14^	Childhood- and adolescent-onset CD	40:0	Angry, sad, and neutral face processing	9/40 Had comorbid ADHD
Rubia *et al*. (2010)^15^	Childhood-onset CD	14:0	Switch task	None
Crowley *et al*. (2010)^16^	CD and substance use disorders	20:0	Risk taking task	Not assessed with *DSM-IV* criteria
Finger *et al*. (2011)^17^	Disruptive behavior disorders + CU	9:6	Passive avoidance learning	10/15 Had comorbid ADHD
Marsh *et al*. (2011)^18^	Disruptive behavior disorders + CU	8:6	Implicit association test with moral words	9/14 Had comorbid ADHD
White *et al*. (2012a)^19^	Disruptive behavior disorders + CU	13:4	Spatial attention with eye gaze cues	9/17 Had comorbid ADHD
White *et al*. (2012b)^20^	Disruptive behavior disorders + CU	12:3	Face processing under high or low attentional load	8/15 Had comorbid ADHD
Sebastian *et al*. (2012)^21^	Conduct problems with or without CU	31:0	Affective theory of mind task	Not assessed with *DSM-IV* criteria; elevated anxiety and depression scores
Viding *et al*. (2012)^22^	Conduct problems with or without CU	30:0	Subliminal face processing task	Not assessed with *DSM-IV* criteria; elevated anxiety and depression scores
White *et al*. (2013)^23^	Disruptive behavior disorders + CU	17:3	Passive avoidance task	4/20 Had comorbid ADHD
Marsh *et al*. (2013)^24^	Disruptive behavior disorders + CU	8:6	Empathy for pain task	8/14 Had comorbid ADHD
Lockwood *et al*. (2013)^25^	Conduct problems with or without CU	37:0	Empathy for pain task	Not assessed with *DSM-IV* criteria; elevated anxiety and depression scores
Sebastian *et al*. (2014)^26^	Conduct problems with or without CU	34:0	Processing emotional faces and eyes	Not Assessed with *DSM-IV* criteria; elevated anxiety and depression scores

Note: ADHD = attention deficit/ hyperactivity disorder; IAPS = International Affective Picture System.
